# Physical health screening for people with severe mental disorders: a best practice implementation project

**DOI:** 10.1590/1980-220X-REEUSP-2025-0370en

**Published:** 2026-03-23

**Authors:** Carlos Alberto dos Santos Treichel, Ana Laura Salomé Lourencetti, Bruno Marinho da Silva, Thaiane Bruna da Cruz Diomedece, Vilanice Alves de Araújo Püschel

**Affiliations:** 1 Universidade de São Paulo, Escola de Enfermagem, Departamento de Enfermagem Materno-Infantil e Psiquiátrica, São Paulo, SP, Brazil.; 2 Centro Brasileiro para o Cuidado à Saúde Baseado em Evidências - JBI Brasil, São Paulo, SP, Brazil.

**Keywords:** Mental Health, Mental Disorders, Mental Health Services, Delivery of Health Care, Integrated, Comorbidity, Saúde Mental, Transtornos Mentais, Serviços de Saúde Mental, Atenção à Saúde, Comorbidade

## Abstract

**Objective:**

To improve physical health screening and assessment practices for individuals with severe and persistent mental disorders treated at a Psychosocial Care Center in Brazil.

**Method:**

A best practice implementation project following the JBI Evidence Implementation Framework conducted in the municipality of Itatiba in the state of São Paulo, with users of the Psychosocial Care Center II. The baseline audit (n = 278) and follow-up audit (n = 134) assessed compliance with seven evidence-based criteria. The data collection process occurred in different phases, which included identifying the area of practice to be changed, engaging change agents, assessing the context and readiness for change, auditing current service practices, implementing practice changes, evaluating the implemented strategies, and monitoring changes in practice.

**Results:**

Baseline compliance was low, with several indicators below 10%. After implementing standardized documentation tools, integrating nursing assessments at intake, and planning inter-service coordination, key improvements were observed. Documentation of physical examination increased from 9.0% to 47.1% (+38.1 pp), Body Mass Index recording from 0% to 22.4% (+22.4 pp), and scheduling of follow-up appointments from 1.1% to 21.6% (+20.5 pp). However, minimal or no progress occurred in laboratory testing, electrocardiogram requests, and monitoring of antipsychotic side effects.

**Conclusion:**

The project strengthened internal routines and demonstrated the feasibility of implementing evidence-based practices. Nevertheless, limited progress in criteria dependent on external factors and the need for stronger professional integration highlight the importance of greater investment to address persistent care gaps.

## RESUMO

**Objetivo:**

Melhorar as práticas de triagem e avaliação da saúde física de indivíduos com transtornos mentais graves e persistentes tratados em um Centro de Atendimento Psicossocial no Brasil.

**Método:**

Projeto de implementação de melhores práticas seguindo a Estrutura de Implementação de Evidências da JBI realizado no município de Itatiba, no estado de São Paulo, com usuários do Centro de Atendimento Psicossocial II. A auditoria inicial (n = 278) e a auditoria de acompanhamento (n = 134) avaliaram a conformidade com sete critérios baseados em evidências. O processo de coleta de dados ocorreu em diferentes fases, que incluíram a identificação da área de prática a ser alterada, o envolvimento de agentes de mudança, a avaliação do contexto e da prontidão para a mudança, a auditoria das práticas atuais de serviço, a implementação de mudanças na prática, a avaliação das estratégias implementadas e o monitoramento das mudanças na prática.

**Resultados:**

A conformidade inicial foi baixa, com vários indicadores abaixo de 10%. Após a implementação de ferramentas de documentação padronizadas, a integração das avaliações de enfermagem na admissão e o planejamento da coordenação entre serviços, foram observadas melhorias significativas. A documentação do exame físico aumentou de 9,0% para 47,1% (+38,1 pp), o registo do Índice de Massa Corporal de 0% para 22,4% (+22,4 pp) e o agendamento de consultas de acompanhamento de 1,1% para 21,6% (+20,5 pp). No entanto, houve um progresso mínimo ou nenhum progresso nos exames laboratoriais, nos pedidos de eletrocardiogramas e na monitorização dos efeitos secundários dos antipsicóticos.

**Conclusão:**

O projeto fortaleceu as rotinas internas e demonstrou a viabilidade da implementação de práticas baseadas em evidências. No entanto, o progresso limitado em critérios dependentes de fatores externos e a necessidade de uma integração profissional mais forte destacam a importância de um maior investimento para lidar com as lacunas persistentes nos cuidados de saúde.

## INTRODUCTION

People with severe and persistent mental disorders (SPMD), such as schizophrenia, bipolar disorder and major depressive disorder with psychotic features, can expect to live 10 to 20 years less than the general population. This disparity is primarily due to preventable and treatable physical health conditions such as cardiovascular disease, type 2 diabetes and respiratory illnesses^(1,2)^. Although factors such as suicide and accidents also contribute to the reduction in life expectancy, there is a growing consensus that physical morbidity and inadequate management of chronic health conditions are the main reasons for this disparity^(3)^.

The literature suggests that disparities in physical health among individuals with SPMD result from the interaction of various personal, professional and organizational factors. At an individual level, cognitive decline, substance use, difficulty recognizing physical symptoms and the adverse metabolic effects of psychotropic medications are particularly relevant^(4)^. From the perspective of mental health professionals, psychiatric issues are often prioritized over the assessment and management of physical conditions, and communication with primary health care providers can be difficult. Primary healthcare professionals, in turn, often exhibit stigmatizing attitudes towards individuals with mental health conditions, which negatively impacts the quality of care provided^(5)^. At an organizational level, failures in coordination between the various components of the health network and a lack of clearly defined responsibilities for managing the physical health of mental health service users exacerbate this issue^(2)^.

In Brazil, a Psychosocial Care Network (RAPS) was formally established by the Ministry of Health in Ordinance No. 3,088/2011. Community mental health policies there have emphasized territorial and intersectoral approaches to care, seeking to strengthen community-based and networked responses^(6)^. However, despite this orientation and the widespread recognition of the importance of integrated care, systematic strategies for physical health screening and assessment within specialized mental health services remain scarce^(7)^. National studies have shown that basic practices such as measuring vital signs, recording body weight and screening for cardiovascular risk factors are implemented inconsistently or not at all in many services^(8)^.

A previous survey conducted during the implementation of Matrix Support in the municipality of Itatiba, São Paulo State, Brazil, revealed that the physical health of users with SPMD was inadequately monitored within specialized services. Although professionals and managers recognized the problem, no structured intervention had been developed to address it at that time. The survey data also showed poor coordination between these users and primary health care services, limiting access to assessments and treatment for chronic clinical conditions^(9)^.

In this context, it is essential to strengthen screening, assessment, and referral processes for physical health within community-based mental health services. International evidence shows that initiatives developed collaboratively with services, managers and professionals to implement best practices can enhance the quality of care and improve coordination across health networks^(10,11)^. However, to our knowledge, no published Brazilian studies have reported on the structured implementation of initiatives specifically designed to improve these practices within community mental health settings.

Based on this evidence and local needs, the present study aimed to improve physical health screening and assessment practices for individuals with severe and persistent mental disorders who are treated at a Psychosocial Care Centre. This is a specialized mental health service within Brazil’s Unified Health System (SUS) that provides community-based care for this population^(12)^, and is located in the municipality of Itatiba in the state of São Paulo. The specific objectives were: (1) assessing the conformity of existing practices with the best clinical recommendations through a baseline clinical audit; (2) identifying barriers and facilitators to improving practice conformity, and implementing strategies to address areas of non-conformity; and (3) evaluating the impact of the implemented strategies through a follow-up audit.

## METHOD

### Study Design

This implementation project follows the JBI Evidence Implementation Framework^(13)^. The JBI approach relies on a structured audit and feedback cycle supported by tools such as the Practical Application of Clinical Evidence System (PACES) and the Getting Research into Practice (GRiP) tool. These instruments are used to gather data and identify barriers to the adoption of best practices^(13)^. The framework is organized into seven distinct phases, outlined in the data collection section.

### Local and Population

The research was conducted among users of the Psychosocial Care Centre II (CAPS II) in the municipality of Itatiba, in the state of São Paulo. The local Psychosocial Care Network (RAPS) comprises 19 primary care services, hospital and emergency care services, and three specialized services: a CAPS II, a CAPS for alcohol and other drugs (CAPS AD) and an outpatient specialist clinic. CAPS II provide community-based care for people with severe and persistent mental health conditions or who are experiencing a mental health crisis. It offers daily support, clinical follow-up and psychosocial care through multidisciplinary teams^(14)^. A total of 278 users met the inclusion criteria and were included in the audit. For the follow-up audit, 134 users were evaluated using the same eligibility criteria. While the two samples partially overlap, they cannot be considered fully paired due to the natural turnover of service users over time (e.g. discharges, new admissions and patients lost to follow-up). Consequently, the follow-up group constitutes a subset of the initial cohort who remained in care and met the inclusion criteria at the second assessment stage.

### Selection Criteria

The eligibility criteria included adults aged 18 years or over who had made at least one recorded visit to the service in the six months prior to data collection.

### Data Collection

#### Phase 1: Identify the Practice Area for Change

The identified problem was the lack of appropriate screening and referral for physical health conditions such as diabetes and hypertension in individuals with mental health conditions who were being treated at CAPS II. These individuals are at an increased risk of physical health problems yet often do not access primary care services or receive comprehensive care within mental health services.

This issue was highlighted in a previous survey conducted in the municipality as part of an evaluation of Matrix Support implementation. The survey revealed a lack of information about physical health in medical records, as well as weak coordination between CAPS II and primary care services. The decision to intervene was made in collaboration with local and municipal managers, who recognized the need to address this care gap^(15)^.

#### Phase 2: Engagement of Change Agents

The project team included the municipal mental health coordinator and the CAPS II coordinator in Itatiba, São Paulo. It also involved psychiatrists, nurses, nursing technicians and primary care professionals. The project was presented as part of a broader strategy to improve coordination between specialized services and primary healthcare, which aligned with the interests of the professionals involved.

#### Phase 3: Context and Readiness Assessment

To assess the context and readiness for change, an in-person meeting was held with CAPS staff and municipal mental health managers. The project team facilitated the session, which included a structured participatory activity using a SWOT (strengths, weaknesses, opportunities, threats) matrix. First, participants were introduced to the goals of the implementation project and the importance of understanding contextual determinants for successful evidence-based practice.

Attendees were then divided into small, mixed-role groups to ensure diverse perspectives. Each group collaboratively discussed and recorded factors that could influence the implementation process, categorizing them as internal or external. These responses were then presented and discussed in a plenary session, allowing for refinement and consensus building. The final matrix was compiled by the project team and shared with all participants for validation. This step provided a structured yet flexible approach to exploring organizational dynamics and gauging the team’s openness to change.

#### Phase 4: Audit of Current Practice (Baseline)

A baseline audit was conducted to evaluate the degree of alignment between current practices and evidence-based recommendations for physical healthcare among CAPS II users in Itatiba, São Paulo. The audit was designed as a cross-sectional descriptive study based on a review of medical records. Data collection took place in December 2024. Eligible participants were adults aged 18 years or older who had visited the service at least once in the six months prior to data collection. A total of 278 users met these criteria and were included in the audit.

Data were collected using a standardized audit form developed by the project team. Two evaluators were trained in advance to ensure consistency in the review process. The audit criteria were based on best practice recommendations identified through a JBI evidence summary and a review of literature, as well as international clinical guidelines^(16)^. Each criterion focused on the documentation of specific elements related to physical health assessment and monitoring. Details of the audit criteria, the target population for each indicator and the methods used to assess compliance are presented in Table 1.

**Table 1:** Audit criteria, sample size and method of measuring compliance – Itatiba, São Paulo, Brazil, 2025.

Audit criterion	Sample	Method used to measure percentage compliance with best practice
1. A physical health examination is conducted and recorded by a healthcare professional.	All users enrolled in the service	Medical records are considered present if a physical health history was documented during the user’s intake.
2. The patient’s medical history is recorded, including information on health behaviors such as smoking status, illicit substance use, alcohol consumption, diet, sleep and physical activity.	All users enrolled in the service for at least 30 days who had at least one visit in the last 6 months	Medical records were present if a user’s health behavior history (including smoking, illicit substance use, alcohol consumption, dietary habits, sleep patterns and physical activity levels) had been documented within the previous six months.
3. Side effects of antipsychotic medication are monitored.	All users enrolled in the service for at least 30 days who had at least one visit in the last 6 months and were prescribed antipsychotic medications	Medical records are up to date if monitoring of side effects has been documented within the last six months, whether they are present or not.
4. Follow-up appointments are scheduled after the initial physical health examination.	All users enrolled in the service for at least 30 days who had at least one visit in the last 6 months	Medical records are considered up to date if a follow-up physical health history was scheduled within the last six months.
5. A fasting blood test is performed and documented to assess thyroid and liver function, blood count and fasting glucose levels.	All users enrolled in the service for at least 30 days who had at least one visit in the last 6 months	Medical records are considered up to date if a fasting blood test to assess thyroid and liver function, blood count and glucose levels was documented in the past year.
6. An electrocardiogram is performed and documented.	All users enrolled in the service for at least 30 days who had at least one visit in the last 6 months	Medical records are present if an electrocardiogram has been documented within the past year.
7. Height and weight are used as a proxy for metabolic risk in non-compliant and/or aggressive patients who refuse blood testing.	All users enrolled in the service for at least 30 days who had at least one visit in the last 6 months	Medical records are up to date if height, weight or body mass index data has been recorded in the past six months.

A descriptive analysis was conducted and the absolute and relative frequencies were calculated for each criterion to establish the baseline level of adherence.

#### Phase 5: Implementation of Practice Changes (GRIP)

The project team evaluated the baseline audit results and discussed them with CAPS II staff and municipal mental health managers. The aim of this collaborative process was to identify the main barriers to complying with the best practice recommendations relating to the physical healthcare of mental health service users. These barriers were explored through structured discussions, drawing on the knowledge of frontline professionals and the experience of the data collectors.

Based on these, the team designed a set of implementation strategies tailored to the specific challenges identified, with the aim of supporting the integration of physical health monitoring into routine care. Strategies were selected through consensus and were explicitly guided by the Expert Recommendations for Implementing Change (ERIC) taxonomy^(17)^. The development process followed the GRiP framework to ensure coherence between the identified barriers, the selected strategies and the expected outcomes. The CAPS II team was provided with feedback on the audit findings and planned strategies to reinforce engagement and prepare for the implementation phase.

#### Phase 6: Follow-up Audit

The follow-up audit used the same methodology, evidence-based audit criteria and data collection procedures as the baseline audit. The aim was to evaluate the impact of the strategies implemented to enhance physical healthcare practices at CAPS II. Data for the follow-up audit was collected in March 2025 from a sample of 134 users who met the same eligibility criteria as in the baseline phase. The same standardized data collection form and procedures were used to ensure comparability.

The follow-up audit findings were then compared with the baseline results to evaluate changes in compliance with best practice criteria. These results were shared with the CAPS II team and municipal mental health managers to inform future improvement efforts and sustain the implemented changes.

#### Phase 7: Ensuring the Sustainability of Practice Changes

The future execution of the planned but not yet implemented strategies will ensure sustainability. Additionally, a new round of data evaluation has been scheduled to monitor the consolidation of changes. The local team will be responsible for ongoing monitoring with technical and academic support, as agreed with service coordination.

### Data Analysis And Treatment

The baseline audit used descriptive analysis to calculate the absolute and relative frequencies of each criterion, establishing the baseline level of adherence. The follow-up audit findings were then compared with the baseline results to evaluate changes in compliance with the best practice criteria.

### Ethics Procedures

The study was approved by the Research Ethics Committee at the University of São Paulo’s School of Nursing and was exempt from the requirement for informed consent (ICF), in accordance with Resolution No. 466/2012 of the Brazilian National Health Council. All necessary precautions were taken to ensure user anonymity.

## RESULTS

### Baseline Audit

The baseline audit revealed low compliance with all seven evidence-based criteria (n = 278). For Criterion 1 (a documented physical health examination at intake), compliance was 9% (n = 25). For Criterion 2 (health behavior history including smoking, alcohol consumption, drug use, diet, sleep and physical activity), compliance was 4.3% (n = 12). For Criterion 3 (monitoring of antipsychotic side effects), compliance was 19.2% (n = 52). For Criterion 4 (scheduling follow-up appointments), compliance was 1.1% (n = 3). For Criterion 5 (fasting blood test in the past year), compliance was 1.4% (n = 4). For criterion 6 (documented electrocardiogram in the past year), compliance was 0.7% (n = 2). For criterion 7 (recorded weight, height, or body mass index in the past six months), no records were found, indicating 0% compliance (n = 0).

### Implementation of Changes to Practice (GRIP)

The project team evaluated the results of the baseline audit and, in collaboration with CAPS II managers and staff, identified the main obstacles to adopting best practices. Strategies were then developed to overcome these barriers and support the implementation of physical health monitoring for users of mental health services. A summary of the barriers, strategies and implementation plans is presented in Table 2.

**Table 2:** A summary of the barriers and strategies employed in the implementation effort, as well as the implementation strategies – Itatiba, São Paulo, Brazil, 2025.

	Lack of standardized documentation	Work overload	Inadequate collaboration between services	Stigma towards mental health
Selected strategy	Change in the record-keeping system	Review of professional roles	Conduct local consensus meetings	Audit and feedback; educational meetings
Actor(s)	Service managers	Service managers and staff	Service managers and staff	Implementation team and network managers
Action	Introduce a standardized stamp for nursing triage/intake assessments	Establish routine nursing assessments at all intake visits	Organize meetings to define workflows with other health services	Conduct feedback sessions and educational activities with other services
Action goal	Increase documentation of physical health information	Increase documentation of physical health information	Improve articulation with physical health and diagnostic services	Reduce stigma and promote integrated care
Timing	Pre-implementation	Pre-implementation	Planned	Planned
Dose	Continuous	Continuous	To be defined	To be defined
Target implementation outcome	Adoption	Adoption	Adoption	Adoption
Justification	Need to standardize health history collection	Need to integrate new practice into routine tasks	Lack of coordination between CAPS and other health services;	Resistance to integrating mental and physical health
Status	Completed	Completed	To be defined	To be defined

#### Barrier 1: Lack of Standardized Documentation Practices

The lack of a standardized format for recording physical health information resulted in inconsistent documentation across users’ charts. To address this issue, service managers introduced a standardized stamp to be used during nursing triage and intake assessments. The stamp included fields for the date and time, blood pressure, weight, height, temperature and date of the last primary care appointment, as well as educational level—an item that had previously been identified as frequently missing from service records. By serving as a visual prompt, the stamp aimed to ensure the systematic documentation of key physical health indicators. This strategy was implemented prior to the follow-up audit and has remained in continuous use with the aim of increasing the adoption of routine documentation practices.

#### Barrier 2: Work Overload Among Caps Staff

Due to the high demand on staff time and competing clinical responsibilities, the systematic assessment of physical health indicators was not consistently prioritized. In response, managers and staff agreed to make routine nursing assessments part of every user intake visit. This change aimed to integrate physical health assessments into existing workflows, thereby reducing the need for additional appointments and facilitating the adoption of this new practice without placing an excessive burden on staff.

#### Barrier 3: Inadequate Collaboration Between Mental Health Services and Diagnostic/Physical Health Services

Although it was not implemented during the reporting period, the team identified the need to promote better coordination with diagnostic and primary care services. To address this issue, a local consensus process has been proposed and will be initiated in the next implementation cycle.

#### Barrier 4: Stigma Towards People with Mental Health Conditions

This barrier was identified as a significant obstacle to integrating physical and mental healthcare. Two strategies were proposed to address stigma within the wider healthcare network: (1) conducting audit and feedback sessions with external services to raise awareness of gaps in care, and (2) organizing educational meetings focused on the physical health needs of people with mental health conditions. These actions are planned for future phases of the project.

### Facilitators: Team Engagement and External Support

The implementation process was facilitated by strong engagement from the local team, active support from municipal health managers, and collaboration with the academic partner institution. Ongoing communication and joint planning increased staff motivation and ownership of the changes. Implementation efforts were supported by regular feedback, on-site discussions and adapting strategies based on team input. These activities were designed to increase the acceptability and feasibility of the proposed changes while maintaining alignment with local practice constraints.

### Follow-up Audit

The follow-up audit, which was conducted with users who had subsequent consultations after the initial assessment (n = 134), revealed some modest improvements in certain criteria, but also identified persistent gaps in others. Compliance with Criterion 1 (documented physical health examination at intake) increased from 9% (n = 25) to 47.1% (n = 63). Criterion 2 (documentation of health behavior history, including smoking, alcohol consumption, drug use, diet, sleep and physical activity) showed compliance of only 4.5% (n = 6). Criterion 3 (monitoring of antipsychotic side effects) was met in only 6.1% of cases (n = 8). Compliance with Criterion 4 (scheduling of follow-up appointments) increased to 20.5%; it was documented in 21.6% of cases (n = 29). Criterion 5 (fasting blood test in the past year) and Criterion 6 (electrocardiogram in the past year) both showed no improvement, with 0% compliance (n = 0). Criterion 7 (documented weight, height, and body mass index in the past six months) showed improvement, reaching 22.4% (n = 30). Table 3 presents compliance with the seven evidence-based audit criteria at baseline and follow-up.

**Table 3:** Summary of compliance with evidence-based criteria at baseline and follow-up – Itatiba, São Paulo, Brazil, 2025.

Criterion	Baseline (n/N, %)	Follow-up (n/N, %)	Δ (p.p.)*
1. A documented physical examination at intake.	25/278 (9.0%)	63/134 (47.1%)	+38.1
2. Documentation of health behavior history (e.g. smoking, alcohol consumption, drug use, diet, sleep and physical activity).	12/278 (4.3%)	6/134 (4.5%)	+0.2
3. Monitoring of antipsychotic side effects.	52/270 (19.2%)	8/131 (6.1%)	–13.1
4. Scheduling of follow-up appointments.	3/278 (1.1%)	29/134 (21.6%)	+20.5
5. Fasting blood test within the past year.	4/278 (1.4%)	0/134 (0.0%)	–1.4
6. A documented electrocardiogram in the past year.	2/278 (0.7%)	0/134 (0.0%)	–0.7
7. Recorded weight, height and/or body mass index in the past six months.	0/278 (0.0%)	30/134 (22.4%)	+22.4

*: Δ (p.p.) = Difference in percentage points between the baseline and the follow-up. **: For Criterion 3, there was a slight difference in the eligible population (baseline N = 270; follow-up N = 131) due to the prescription of antipsychotic medication.

## DISCUSSION

The aim of this implementation project was to align the physical health screening and assessment practices at a Brazilian community mental health center with the best practice recommendations. The aim was to enhance the identification, documentation and management of physical health conditions among individuals with SPMD, who are known to experience disproportionately high rates of preventable illnesses and premature mortality^(18,19)^. By integrating evidence-based physical healthcare processes into routine mental health services, the project aimed to address a significant shortcoming that has been widely documented in national and international literature.

The baseline audit confirmed this gap. Compliance with all seven evidence-based criteria was alarmingly low, ranging from 0% to 20.3%. These findings are consistent with prior research showing that people with SPMD often receive inadequate physical healthcare due to systemic fragmentation, low prioritization of physical health in psychiatric settings and provider-level barriers such as stigma, role ambiguity and lack of training^(5,20,21)^. In this case, the audit served to quantify the extent of the problem, raise awareness among staff, and provide a concrete starting point for intervention.

Following the implementation of targeted strategies, the follow-up audit revealed meaningful improvements in three of the prioritized indicators. The percentage of patients receiving a physical health assessment at intake increased from 9% to 47.1% (Criterion 1); the percentage of patients with follow-up appointments scheduled rose from 1.1% to 21.6% (Criterion 4); and the percentage of patients with weight, height, or Body Mass Index documented improved from 0% to 22.4% (Criterion 7). These results highlight the potential of simple, pragmatic strategies to improve the quality of care in environments with limited resources.

In particular, introducing a standardized stamp during intake assessments enabled nurses to systematically document physical health parameters as part of their established workflow. The reconfiguration of professional roles to involve nurses more actively in physical assessments also proved effective in promoting team-based care. Improvements in follow-up scheduling suggest the emergence of a culture of continuity of care—a critical step in the long-term monitoring and management of chronic conditions which is often overlooked in psychiatric services. Similarly, increased documentation of anthropometric data is a fundamental yet vital approach to identifying individuals at risk of metabolic complications, particularly when access to laboratory tests is limited^(22)^.

These improvements are consistent with the wider body of literature on implementation science, which has demonstrated that interventions that are co-designed and operationalized by frontline workers tend to be more acceptable, feasible and sustainable^(23,24)^. When staff are involved in defining problems and solutions, they are more likely to take ownership of changes, adapt them to local circumstances and ensure they are adhered to in day-to-day practice^(23)^.

Nevertheless, progress varied across the criteria. Two indicators showed little or no improvement. Criterion 2 (comprehensive documentation of health behavior history) increased from 4.3% to only 4.5%. This indicator required the recording of multiple health behaviors, such as tobacco use, alcohol consumption, illicit drug use, diet, sleep and physical activity. In many cases, however, only a subset of these behaviors was documented. This suggests that the scope of the indicator may have exceeded what was feasible under existing documentation routines. This highlights the need for standardized tools or checklists to facilitate the collection of comprehensive data without overburdening staff.

The percentage of patients for whom antipsychotic side effects were monitored actually declined, from 19.2% to 6.1%. This drop may be due to the limited involvement of prescribing physicians in the implementation process and the absence of standardized formats or prompts for recording side effect information. It is possible that physicians chose not to document anything when no side effects were reported; however, best practice emphasizes that monitoring should be recorded systematically, regardless of whether adverse effects are observed^(25)^. Addressing this issue will require strategies that are more tailored to strengthen physician engagement and accountability in implementation initiatives. This could involve co-developing standardized monitoring forms, incorporating prompts into clinical records, conducting targeted feedback sessions for prescribers and linking antipsychotic monitoring to multidisciplinary care plans.

There was no improvement for criteria 5 (laboratory testing, declining from 1.4% to 0%) and 6 (electrocardiogram performance, declining from 0.7% to 0%), both of which rely heavily on external services. These procedures involve multiple logistical steps, including issuing test requests, scheduling and attending appointments at municipal laboratories, retrieving results and returning to the CAPS for follow-up. Given the short timeframe between audits, it is unsurprising that no significant change was observed for these indicators. However, this highlights a broader structural issue already documented in the literature: limited integration between community mental health services and the wider health system severely constrains the feasibility of timely, coordinated care^(23)^. Without interoperable systems, clearer referral pathways and improved access to services, even well-planned internal initiatives may fall short of their potential impact^(26,27)^.

The lack of improvement in indicators dependent on external services highlights that barriers to physical healthcare can extend beyond the scope of specialized mental health services. In this context, another important consideration is how the different parts of the healthcare network are connected. Difficulties in integrating services can lead to the fragmentation of the care network, limiting the provision of longitudinal and comprehensive care^(28)^. One study showed that managers, healthcare professionals and patients identified the lack of clearly defined care pathways, limited knowledge of the network’s components and access criteria, and difficulties in case communication and discharge from specialist services as major barriers^(28)^. In this context, shared care models such as matrix support encourage collaboration between services and promote an integrated and coordinated approach to addressing mental and physical health needs^(29)^.

Some limitations should be considered. Firstly, the relatively short timeframe between the baseline assessment in December 2024 and the follow-up assessment in March 2025 may not have allowed for the complete implementation and consolidation of the changes. The observed improvements may partially reflect the Hawthorne effect rather than sustained behavioral change. Secondly, data on process and fidelity measures, such as the proportion of assessments for which the standardized stamp was used, adherence to documentation practices among professionals, and the number of feedback meetings conducted, were not systematically recorded. The absence of these indicators limits the ability to interpret the implementation process and its mechanisms of change more precisely. Thirdly, the smaller sample size in the follow-up audit, resulting from user turnover and variations in service flow, may affect comparability. Finally, as the evaluation relied solely on medical record review, actual changes in clinical practice may have been underestimated when documentation was incomplete or delayed. Such limitations are common in real-world quality improvement initiatives, however, and highlight the importance of including process evaluation and triangulating data sources in future studies.

Despite these constraints, the project generated actionable insights for improving physical health monitoring in community mental health services in Brazil and other similar contexts. Our results demonstrate that team-driven interventions that are adapted to local workflows and supported by visual aids or simple tools are feasible and effective. Future efforts should prioritize inter-service integration, enhance the consistency of clinical documentation and incorporate structured audit and feedback cycles into routine practice.

Furthermore, including user perspectives in studies that assess long-term outcomes could deepen our understanding of how improved screening translates into better physical and mental health. Ultimately, meeting the physical health needs of people with severe mental illness requires sustained institutional commitment, shared accountability and a broader vision of integrated, equitable care, as well as technical solutions^(27)^.

## CONCLUSION

This project demonstrated that incorporating simple, low-cost strategies into routine workflows can meaningfully enhance physical health screening practices within community mental health settings. The introduction of standardized documentation tools and the redefinition of professional roles—particularly the increased involvement of nursing staff—led to significant improvements in key areas such as physical assessments, anthropometric measurements and follow-up appointment scheduling. These changes signal early progress towards integrating physical and mental healthcare.

However, the project also highlighted the limitations of partial team engagement and structural fragmentation, as indicators dependent on physician participation or external service coordination showed little to no improvement. Future initiatives should build on these lessons by fostering inclusive planning, reinforcing interprofessional collaboration and addressing systemic barriers to continuity of care. Practical next steps include strengthening the link between CAPS II, primary care and laboratory services to facilitate follow-up of clinical parameters, implementing standardized checklists for monitoring antipsychotic side effects, and integrating electronic reminders or prompts into health records to support routine documentation. Sustained improvements will also depend on the institutionalization of audit and feedback mechanisms, as well as ongoing professional development. By illustrating the potential and challenges of improving physical healthcare within psychosocial care settings, this study provides actionable insights for healthcare systems aiming to reduce the mortality gap experienced by individuals with severe mental health conditions.

## DATA AVAILABILITY

The entire dataset supporting the results of this study is available upon request to the corresponding author.
