# Supplementary material for: Physical health screening for people with severe mental disorders: a best practice implementation project
Source: Rev Esc Enferm USP. 2026 Mar 23;60:e20250370. doi: 10.1590/1980-220X-REEUSP-2025-0370en (PMC13020551; doi:10.1590/1980-220X-REEUSP-2025-0370en)
Supplement: Translation

# Translation: Seguimiento de la salud física de personas con trastornos mentales graves: proyecto de implementación de prácticas mejoradas

**DOI:** 10.1590/1980-220X-REEUSP-2025-0370es

**Authors:** Carlos Alberto dos Santos Treichel, Ana Laura Salomé Lourencetti, Bruno Marinho da Silva, Thaiane Bruna da Cruz Diomedece, Vilanice Alves de Araújo Püschel

**Affiliations:** 1 Universidade de São Paulo, Escola de Enfermagem, Departamento de Enfermagem Materno-Infantil e Psiquiátrica, São Paulo, SP, Brasil.; 2 Centro Brasileiro para o Cuidado à Saúde Baseado em Evidências - JBI Brasil, São Paulo, SP, Brasil.

**Keywords:** Salud Mental, Trastornos Mentales, Servicios de Salud Mental, Atención a la Salud, Comorbilidad

## Abstract

**Objetivo:**

Mejorar las prácticas de triaje y evaluación de la salud física de personas con trastornos mentales graves y persistentes asistidas en un centro de atención psicosocial de Brasil.

**Método:**

Se trata de un proyecto para establecer prácticas mejoradas según la Estructura de Implementación de Evidencias de la JBI, que se realizó en el municipio de Itatiba, estado de São Paulo, con usuarios del Centro de Atención Psicosocial II. La auditoría inicial (n = 278) y la auditoría de seguimiento (n = 134) evaluaron el cumplimiento de siete criterios basados en la evidencia. El proceso de recopilación de datos se llevó a cabo en diferentes fases, a saber, la identificación del área de la práctica a ser alterada, la participación de agentes de cambio, la evaluación del contexto y de la disposición al cambio, la auditoría de las prácticas usuales del servicio, la implementación de cambios en la práctica, la evaluación de las estrategias implementadas y el monitoreo de los cambios en la práctica.

**Resultados:**

La consecución inicial fue baja, con varios indicadores que no alcanzaban el 10%. Tras la implementación de herramientas de documentación estandarizadas, la integración de las evaluaciones de enfermería en el ingreso y la planificación de la coordinación entre servicios, se observaron mejoras significativas. La documentación del examen físico aumentó del 9,0% al 47,1% (+38,1 pp), el registro del Índice de Masa Corporal, del 0% al 22,4% (+22,4 pp) y la programación de consultas de seguimiento, del 1,1% al 21,6% (+20,5 pp). Sin embargo, se observó un progreso mínimo o nulo en los exámenes de laboratorio, en las solicitudes de electrocardiogramas y en la monitorización de los efectos secundarios de los antipsicóticos.

**Conclusión:**

El proyecto fortaleció las rutinas internas y demostró la viabilidad de implementar prácticas basadas en la evidencia. No obstante, el progreso limitado en criterios dependientes de factores externos y la necesidad de una integración profesional más sólida resaltan la importancia de una inversión mayor para abordar las brechas persistentes en la atención médica.

## INTRODUCCIÓN

Las personas con trastornos mentales graves y persistentes (TMGP), como la esquizofrenia, el trastorno bipolar y el trastorno depresivo mayor con características psicóticas, pueden esperar vivir entre 10 y 20 años menos que la población general. Esta disparidad se debe principalmente a afecciones de salud física prevenibles y tratables, como las enfermedades cardiovasculares, la diabetes tipo 2 y las enfermedades respiratorias^(1,2)^.

Aunque factores como el suicidio y los accidentes también contribuyen a la reducción de la esperanza de vida, existe un consenso cada vez mayor en que la morbilidad física y el manejo inadecuado de las afecciones crónicas son las principales razones de esta disparidad^(3)^.

La literatura sugiere que las disparidades en la salud física entre las personas con TMGP son el resultado de la interacción de diversos factores personales, profesionales y organizativos. A nivel individual, el deterioro cognitivo, el consumo de sustancias, la dificultad para reconocer los síntomas físicos y los efectos metabólicos adversos de los medicamentos psicotrópicos son especialmente relevantes^(4)^. Desde la perspectiva de los profesionales de la salud mental, a menudo se da prioridad a los problemas psiquiátricos sobre la evaluación y el manejo de las afecciones físicas, y la comunicación con los proveedores de atención de salud primaria puede resultar difícil. A su vez, los profesionales de la atención de salud primaria suelen mostrar actitudes estigmatizantes hacia las personas con trastornos de salud mental, lo que repercute negativamente en la calidad de la atención prestada^(5)^. A nivel organizativo, la falta de coordinación entre los distintos componentes de la red de atención de salud y la falta de responsabilidades claramente definidas para gestionar la salud física de los usuarios de los servicios de salud mental agravan este problema^(2)^.

En Brasil, el Ministerio de Salud estableció formalmente una Red de Atención Psicosocial (RAPS) mediante la Ordenanza n.º 3088/2011. Las políticas comunitarias de salud mental han hecho hincapié en los enfoques territoriales e intersectoriales de la atención, con el fin de reforzar las respuestas comunitarias y en red^(6)^. Sin embargo, a pesar de esta orientación y del reconocimiento generalizado de la importancia de la atención integrada, siguen siendo escasas las estrategias sistemáticas de detección y evaluación de la salud física en los servicios especializados de salud mental^(7)^. Estudios nacionales han demostrado que prácticas básicas como la medición de los signos vitales, el registro del peso corporal y la detección de factores de riesgo cardiovascular se aplican de forma inconsistente o no se aplican en absoluto en muchos servicios^(8)^.

Una encuesta previa realizada durante la implementación de Matrix Support (Apoyo por Matrices) en el municipio de Itatiba, estado de São Paulo (Brasil), reveló que la salud física de los usuarios con TMGP no se monitoreaba adecuadamente en los servicios especializados. Aunque los profesionales y los gestores reconocían el problema, en ese momento no se había desarrollado ninguna intervención estructurada para abordarlo. Los datos de la encuesta también mostraron una mala coordinación entre estos usuarios y los servicios de salud, lo que limitaba el acceso a las evaluaciones y al tratamiento de las afecciones clínicas crónicas^(9)^.

En este contexto, es esencial reforzar los procesos de detección, evaluación y derivación de la salud física en los servicios comunitarios de salud mental. Las pruebas internacionales demuestran que las iniciativas desarrolladas en colaboración con los servicios, los gestores y los profesionales para implementar las mejores prácticas pueden mejorar la calidad de la atención de salud y la coordinación entre las redes de salud^(10,11)^. Sin embargo, según nuestro conocimiento, ningún estudio brasileño publicado ha informado sobre la implementación estructurada de iniciativas diseñadas específicamente para mejorar estas prácticas en los entornos comunitarios de salud mental.

Basándose en esta evidencia y en las necesidades locales, el presente estudio tuvo como objetivo mejorar las prácticas de detección y evaluación de la salud física de las personas con trastornos mentales graves y persistentes que son tratadas en un Centro de Atención Psicosocial. Se trata de un servicio especializado de salud mental dentro del Sistema Único de Salud (SUS) de Brasil que proporciona atención comunitaria a esta población^(12)^ y está ubicado en el municipio de Itatiba, en el estado de São Paulo. Los objetivos específicos eran: (1) evaluar la conformidad de las prácticas existentes con las mejores recomendaciones clínicas mediante una auditoría clínica de referencia; (2) identificar las barreras y los facilitadores para mejorar la conformidad de las prácticas, e implementar estrategias para abordar las áreas de no conformidad; y (3) evaluar el impacto de las estrategias implementadas mediante una auditoría de seguimiento.

## MÉTODO

### Diseño del Estudio

Este proyecto de implementación sigue el Marco de Implementación de Evidencia de JBI^(13)^. El enfoque de JBI se basa en un ciclo estructurado de auditoría y retroalimentación respaldado por herramientas como el Sistema de Aplicación Práctica de Evidencia Clínica (*Practical Application of Clinical Evidence System* - PACES) y la herramienta *Getting Research into Practice* (GRiP). Estos instrumentos se utilizan para recolectar datos e identificar barreras para la adopción de las mejores prácticas^(13)^. El marco se organiza en siete fases distintas, que se describen en la sección de recolección de datos.

### Local y Población

La investigación se llevó a cabo entre los usuarios del Centro de Atención Psicosocial II (CAPS II) en el municipio de Itatiba, en el estado de São Paulo. La Red de Atención Psicosocial (RAPS) local comprende 19 servicios de atención primaria, servicios hospitalarios y de urgencias, y tres servicios especializados: un CAPS II, un CAPS para alcohol y otras drogas (CAPS AD) y una clínica especializada ambulatoria. El CAPS II proporciona atención comunitaria a personas con trastornos mentales graves y persistentes o que están atravesando una crisis de salud mental. Ofrece apoyo diario, seguimiento clínico y atención psicosocial a través de equipos multidisciplinarios^(14)^. Un total de 278 usuarios cumplieron los criterios de inclusión y fueron incluidos en la auditoría. Para la auditoría de seguimiento, se evaluó a 134 usuarios utilizando los mismos criterios de elegibilidad. Aunque las dos muestras se solapan parcialmente, no pueden considerarse totalmente emparejadas debido a la rotación natural de los usuarios del servicio a lo largo del tiempo (por ejemplo, altas, nuevas admisiones y pacientes perdidos durante el seguimiento). En consecuencia, el grupo de seguimiento constituye un subconjunto de la cohorte inicial que permaneció en atención y cumplió los criterios de inclusión en la segunda fase de evaluación.

### Criterios de Selección

Los criterios de elegibilidad incluían a adultos de 18 años o más que hubieran realizado al menos una visita registrada al servicio en los seis meses anteriores a la recolección de datos.

### Recolección de Datos

#### Fase 1: Identificar el Área de Práctica Para el Cambio

El problema identificado fue la falta de detección y derivación adecuadas para afecciones de salud física como la diabetes y la hipertensión en personas con afecciones de salud mental que estaban siendo tratadas en CAPS II. Estas personas tienen un mayor riesgo de padecer problemas de salud física, pero a menudo no acceden a los servicios de atención de salud ni reciben una atención integral dentro de los servicios de salud mental.

Esta cuestión se puso de relieve en una encuesta anterior realizada en el municipio como parte de una evaluación de la implementación de Matrix Support. La encuesta reveló una falta de información sobre la salud física en los registros médicos, así como una débil coordinación entre CAPS II y los servicios de atención primaria. La decisión de intervenir se tomó en colaboración con los gestores locales y municipales, que reconocieron la necesidad de abordar esta brecha en la atención^(15)^.

#### Fase 2: Participación de Los Agentes del Cambio

El equipo del proyecto incluía al coordinador municipal de salud mental y al coordinador de CAPS II en Itatiba, São Paulo. También participaron psiquiatras, enfermeros, técnicos de enfermería y profesionales de la atención primaria. El proyecto se presentó como parte de una estrategia más amplia para mejorar la coordinación entre los servicios especializados y la atención de salud, lo que coincidía con los intereses de los profesionales involucrados.

#### Fase 3: Evaluación del Contexto y la Preparación

Para evaluar el contexto y la preparación para el cambio, se celebró una reunión presencial con el personal del CAPS y los responsables municipales de salud mental. El equipo del proyecto facilitó la sesión, que incluyó una actividad participativa estructurada utilizando una matriz DAFO (debilidades, amenazas, fortalezas y oportunidades). En primer lugar, se presentó a los participantes los objetivos del proyecto de implementación y la importancia de comprender los determinantes contextuales para el éxito de la práctica basada en la evidencia.

A continuación, los asistentes se dividieron en pequeños grupos de funciones mixtas para garantizar la diversidad de perspectivas. Cada grupo debatió y registró de forma colaborativa los factores que podían influir en el proceso de implementación, clasificándolos como internos o externos. A continuación, estas respuestas se presentaron y debatieron en una sesión plenaria, lo que permitió perfeccionarlas y alcanzar un consenso. El equipo del proyecto recopiló la matriz final y la compartió con todos los participantes para su validación. Este paso proporcionó un enfoque estructurado pero flexible para explorar la dinámica organizativa y evaluar la apertura del equipo al cambio.

#### Fase 4: Auditoría de la Práctica Actual (Línea de Base)

Se llevó a cabo una auditoría de referencia para evaluar el grado de alineación entre las prácticas actuales y las recomendaciones basadas en la evidencia para la atención médica física entre los usuarios de CAPS II en Itatiba, São Paulo. La auditoría se diseñó como un estudio descriptivo transversal basado en la revisión de los registros médicos. La recolección de datos se llevó a cabo en diciembre de 2024. Los participantes elegibles eran adultos de 18 años o más que habían visitado el servicio al menos una vez en los seis meses anteriores a la recolección de datos. Un total de 278 usuarios cumplían estos criterios y fueron incluidos en la auditoría.

Los datos se recolectaron utilizando un formulario de auditoría estandarizado elaborado por el equipo del proyecto. Se formó previamente a dos evaluadores para garantizar la coherencia en el proceso de revisión. Los criterios de auditoría se basaron en las recomendaciones de buenas prácticas identificadas a través de un resumen de pruebas del JBI y una revisión de la literatura, así como en las directrices clínicas internacionales^(16)^. Cada criterio se centró en la documentación de elementos específicos relacionados con la evaluación y el monitoreo de la salud física. En la Tabla 1 se presentan los detalles de los criterios de auditoría, la población objetivo de cada indicador y los métodos utilizados para evaluar el cumplimiento.

**Tabla 1:** Criterios de auditoría, tamaño de la muestra y método de medición del cumplimiento – Itatiba, São Paulo, Brasil, 2025.

Criterio de auditoría	Muestra	Método utilizado para medir el porcentaje de cumplimiento de las mejores prácticas
1. Un profesional sanitario realiza y registra un examen físico.	Todos los usuarios inscritos en el servicio	Se considera que existen registros médicos si se documentó el historial médico físico durante la admisión del usuario.
2. Se registra el historial médico del paciente, incluida la información sobre hábitos de salud, como el tabaquismo, el consumo de sustancias ilegales, el consumo de alcohol, la dieta, el sueño y la actividad física.	Todos los usuarios inscritos en el servicio durante al menos 30 días que hayan tenido al menos una visita en los últimos 6 meses	Se consideraban presentes los registros médicos si se había documentado el historial de hábitos de salud del usuario (incluido el tabaquismo, el consumo de sustancias ilegales, el consumo de alcohol, los hábitos alimenticios, los patrones de sueño y los niveles de actividad física) en los seis meses anteriores.
3. Se controlan los efectos secundarios de la medicación antipsicótica.	Todos los usuarios inscritos en el servicio durante al menos 30 días que hayan acudido al menos a una visita en los últimos 6 meses y a los que se les hayan recetado medicamentos antipsicóticos.	Los registros médicos están actualizados si se ha documentado el control de los efectos secundarios en los últimos seis meses, independientemente de que estén presentes o no.
4. Se programan citas de seguimiento después del examen físico inicial.	Todos los usuarios inscritos en el servicio durante al menos 30 días que hayan acudido al menos a una visita en los últimos 6 meses	Los registros médicos se consideran actualizados si se programó un historial médico de seguimiento en los últimos seis meses.
5. Se realiza y documenta un análisis de sangre en ayunas para evaluar la función tiroidea y hepática, el recuento sanguíneo y los niveles de glucosa en ayunas.	Todos los usuarios inscritos en el servicio durante al menos 30 días que hayan tenido al menos una visita en los últimos 6 meses	Los registros médicos se consideran actualizados si se ha documentado un análisis de sangre en ayunas para evaluar la función tiroidea y hepática, el recuento sanguíneo y los niveles de glucosa en el último año.
6. Se realiza y documenta un electrocardiograma.	Todos los usuarios inscritos en el servicio durante al menos 30 días que hayan tenido al menos una visita en los últimos 6 meses	Los registros médicos se consideran actualizados si se ha documentado un electrocardiograma en el último año.
7. La altura y el peso se utilizan como indicadores del riesgo metabólico en pacientes no cumplidores y/o agresivos que se niegan a someterse a análisis de sangre.	Todos los usuarios inscritos en el servicio durante al menos 30 días que hayan acudido al menos a una visita en los últimos 6 meses	Los registros médicos están actualizados si se han registrado datos de altura, peso o índice de masa corporal en los últimos seis meses.

Se realizó un análisis descriptivo y se calcularon las frecuencias absolutas y relativas para cada criterio con el fin de establecer el nivel de referencia de cumplimiento.

#### Fase 5: Implementación de Cambios en la Práctica (Getting Research Into Practice – GRIP)

El equipo del proyecto evaluó los resultados de la auditoría de referencia y los discutió con el personal de CAPS II y los responsables municipales de salud mental. El objetivo de este proceso colaborativo era identificar las principales barreras para cumplir con las recomendaciones de buenas prácticas relacionadas con la atención de salud física de los usuarios de los servicios de salud mental. Estas barreras se exploraron a través de debates estructurados, basándose en los conocimientos de los profesionales de primera línea y la experiencia de los recopiladores de datos.

A partir de ellos, el equipo diseñó un conjunto de estrategias de implementación adaptadas a los retos específicos identificados, con el objetivo de apoyar la integración del monitoreo de la salud física en la atención rutinaria. Las estrategias se seleccionaron por consenso y se guiaron explícitamente por la taxonomía de las Recomendaciones de Expertos para la Implementación del Cambio (*Expert Recommendations for Implementing Change* - ERIC)^(17)^. El proceso de desarrollo siguió el marco GRiP para garantizar la coherencia entre los obstáculos identificados, las estrategias seleccionadas y los resultados esperados. Se proporcionó al equipo de CAPS II información sobre los resultados de la auditoría y las estrategias previstas para reforzar el compromiso y preparar la fase de implementación.

#### Fase 6: Auditoría de Seguimiento

La auditoría de seguimiento utilizó la misma metodología, criterios de auditoría basados en la evidencia y procedimientos de recolección de datos que la auditoría inicial. El objetivo era evaluar el impacto de las estrategias implementadas para mejorar las prácticas de atención médica física en CAPS II. Los datos para la auditoría de seguimiento se recolectaron en marzo de 2025 a partir de una muestra de 134 usuarios que cumplían los mismos criterios de elegibilidad que en la fase inicial. Se utilizaron el mismo formulario y los mismos procedimientos estandarizados de recolección de datos para garantizar la comparabilidad.

A continuación, se compararon los resultados de la auditoría de seguimiento con los resultados de referencia para evaluar los cambios en el cumplimiento de los criterios de buenas prácticas. Estos resultados se compartieron con el equipo de CAPS II y los responsables municipales de salud mental para informar sobre los futuros esfuerzos de mejora y mantener los cambios implementados.

#### Fase 7: Garantizar La Sostenibilidad De Los Cambios En Las Prácticas

La futura ejecución de las estrategias planificadas pero aún no implementadas garantizará la sostenibilidad. Además, se ha programado una nueva ronda de evaluación de datos para monitorear la consolidación de los cambios. El equipo local se encargará de la supervisión continua con apoyo técnico y académico, según lo acordado con la coordinación del servicio.

### Análisis y Tratamiento de Datos

La auditoría inicial utilizó un análisis descriptivo para calcular las frecuencias absolutas y relativas de cada criterio, estableciendo el nivel inicial de cumplimiento. A continuación, los resultados de la auditoría de seguimiento se compararon con los resultados iniciales para evaluar los cambios en el cumplimiento de los criterios de buenas prácticas.

### Procedimientos Éticos

El estudio fue aprobado por el Comité de Ética en Investigación de la Facultad de Enfermería de la Universidad de São Paulo y quedó exento del requisito de consentimiento informado (CI), de conformidad con la Resolución n.º 466/2012 del Consejo Nacional de Salud de Brasil. Se tomaron todas las precauciones necesarias para garantizar el anonimato de los usuarios.

## RESULTADOS

### Auditoría Inicial

La auditoría inicial reveló un bajo cumplimiento de los siete criterios basados en la evidencia (n = 278). Para el criterio 1 (un examen físico documentado en el momento de la admisión), el cumplimiento fue del 9% (n = 25). Para el criterio 2 (historial de hábitos de salud, incluyendo tabaquismo, consumo de alcohol, consumo de drogas, dieta, sueño y actividad física), el cumplimiento fue del 4,3% (n = 12). En cuanto al criterio 3 (monitoreo de los efectos secundarios de los antipsicóticos), el cumplimiento fue del 19.2% (n = 52). En cuanto al criterio 4 (programación de citas de seguimiento), el cumplimiento fue del 1.1% (n = 3). En cuanto al criterio 5 (análisis de sangre en ayunas en el último año), el cumplimiento fue del 1.4% (n = 4). En cuanto al criterio 6 (electrocardiograma documentado en el último año), el cumplimiento fue del 0,7% (n = 2). En cuanto al criterio 7 (registro del peso, la estatura o el índice de masa corporal en los últimos seis meses), no se encontraron registros, lo que indica un cumplimiento del 0% (n = 0).

### Implementación de Cambios en la Práctica (Getting Research into Practice – GRIP)

El equipo del proyecto evaluó los resultados de la auditoría inicial y, en colaboración con los directores y el personal de CAPS II, identificó los principales obstáculos para la adopción de las mejores prácticas. A continuación, se desarrollaron estrategias para superar estas barreras y apoyar la implementación del monitoreo de la salud física de los usuarios de los servicios de salud mental. En la Tabla 2 se presenta un resumen de las barreras, las estrategias y los planes de implementación.

**Tabla 2:** Resumen de las barreras y estrategias empleadas en el esfuerzo de implementación, así como las estrategias de implementación – Itatiba, São Paulo, Brasil, 2025.

	Falta de documentación estandarizada	Sobrecarga de trabajo	Colaboración inadecuada entre los servicios	Estigma hacia la salud mental
Estrategia seleccionada	Cambio en el sistema de registro	Revisión de las funciones profesionales	Celebración de reuniones locales para alcanzar un consenso	Auditoría y retroalimentación; reuniones educativas
Actores	Gerentes de servicios	Gerentes y personal de servicios	Gerentes de servicios y personal	Equipo de implementación y gestores de red
Acción	Introducir un sello estandarizado para las evaluaciones de selección/admisión de enfermería	Establecer evaluaciones de enfermería rutinarias en todas las visitas de admisión	Organizar reuniones para definir los flujos de trabajo con otros servicios de salud	Realizar sesiones de retroalimentación y actividades educativas con otros servicios
Objetivo de la acción	Aumentar la documentación de la información sobre la salud física.	Aumentar la documentación de la información sobre salud física	Mejorar la articulación con los servicios de salud física y diagnóstico	Reducir el estigma y promover la atención integrada
Calendario	Antes de la implementación	Pre implementación	Planificado	Planificado
Dosis	Continua	Continuo	Por definir	Por definir
Resultado previsto de la implementación	Adopción	Adopción	Adopción	Adopción
Justificación	Necesidad de estandarizar la recopilación del historial médico	Necesidad de integrar nuevas prácticas en las tareas rutinarias	Falta de coordinación entre CAPS y otros servicios de salud	Resistencia a integrar la salud mental y física
Estado	Completado	Completado	Por definir	Por definir

#### Barrera 1: Falta de Prácticas de Documentación Estandarizadas

La falta de un formato estandarizado para registrar la información sobre la salud física daba lugar a una documentación inconsistente en las historias clínicas de los usuarios. Para solucionar este problema, los administradores del servicio introdujeron un sello estandarizado que se utilizaba durante la clasificación de enfermería y las evaluaciones de admisión. El sello incluía campos para la fecha y la hora, la presión arterial, el peso, la estatura, la temperatura y la fecha de la última cita de atención primaria, así como el nivel educativo, un dato que anteriormente se había identificado como frecuentemente ausente en los registros de los servicios de salud. Al servir como recordatorio visual, el sello tenía por objeto garantizar la documentación sistemática de los indicadores clave de salud física. Esta estrategia se aplicó antes de la auditoría de seguimiento y se ha seguido utilizando con el fin de aumentar la adopción de prácticas de documentación rutinarias.

#### Barrera 2: Sobrecarga de Trabajo del Personal de Caps

Debido a la gran demanda de tiempo del personal y a las responsabilidades clínicas contrapuestas, la evaluación sistemática de los indicadores de salud física no se priorizaba de manera sistemática. En respuesta a ello, los directivos y el personal acordaron incluir evaluaciones de enfermería rutinarias en todas las visitas de admisión de los usuarios. Este cambio tenía por objeto integrar las evaluaciones de salud física en los flujos de trabajo existentes, reduciendo así la necesidad de citas adicionales y facilitando la adopción de esta nueva práctica sin imponer una carga excesiva al personal.

#### Barrera 3: Colaboración Inadecuada Entre Los Servicios de Salud Mental y los Servicios de Diagnóstico/Salud Física

Aunque no se implementó durante el período del informe, el equipo identificó la necesidad de promover una mejor coordinación con los servicios de diagnóstico y atención primaria. Para abordar esta cuestión, se ha propuesto un proceso de consenso local que se iniciará en el próximo ciclo de implementación.

#### Barrera 4: Estigma Hacia Las Personas Con Trastornos de Salud Mental

Este obstáculo se identificó como un impedimento significativo para la integración de la atención de salud física y mental. Se propusieron dos estrategias para abordar el estigma dentro de la red de atención de salud en general: (1) realizar auditorías y sesiones de retroalimentación con servicios externos para crear conciencia sobre las brechas en la atención, y (2) organizar reuniones educativas centradas en las necesidades de salud física de las personas con trastornos de salud mental. Estas acciones están previstas para fases futuras del proyecto.

### Facilitadores: Compromiso del Equipo y Apoyo Externo

El proceso de implementación se vio facilitado por el fuerte compromiso del equipo local, el apoyo activo de los gestores municipales de salud y la colaboración con la institución académica asociada. La comunicación continua y la planificación conjunta aumentaron la motivación del personal y su implicación en los cambios. Los esfuerzos de implementación se vieron respaldados por comentarios periódicos, debates in situ y la adaptación de estrategias basadas en las aportaciones del equipo. Estas actividades se diseñaron para aumentar la aceptabilidad y la viabilidad de los cambios propuestos, manteniendo al mismo tiempo la alineación con las limitaciones de la práctica local.

### Auditoría de Seguimiento

La auditoría de seguimiento, que se llevó a cabo con usuarios que tuvieron consultas posteriores a la evaluación inicial (n = 134), reveló algunas mejoras modestas en ciertos criterios, pero también identificó deficiencias persistentes en otros. El cumplimiento del criterio 1 (examen físico documentado en el momento de la admisión) aumentó del 9% (n = 25) al 47,1% (n = 63). El criterio 2 (documentación del historial de hábitos de salud, incluyendo tabaquismo, consumo de alcohol, consumo de drogas, dieta, sueño y actividad física) mostró un cumplimiento de solo el 4.5% (n = 6). El criterio 3 (monitoreo de los efectos secundarios de los antipsicóticos) se cumplió solo en el 6.1% de los casos (n = 8). El cumplimiento del criterio 4 (programación de citas de seguimiento) aumentó hasta el 20,5%; se documentó en el 21,6% de los casos (n = 29). El criterio 5 (análisis de sangre en ayunas en el último año) y el criterio 6 (electrocardiograma en el último año) no mostraron ninguna mejora, con un cumplimiento del 0% (n = 0). El criterio 7 (peso, altura e índice de masa corporal documentados en los últimos seis meses) mostró una mejora, alcanzando el 22,4% (n = 30). La Tabla 3 presenta el cumplimiento de los siete criterios de auditoría basados en la evidencia al inicio del estudio y en el seguimiento.

**Tabla 3:** Resumen del cumplimiento de los criterios basados en la evidencia al inicio del estudio y durante el seguimiento – Itatiba, São Paulo, Brasil, 2025.

Criterio	Inicio (n/N, %)	Seguimiento (n/N, %)	Δ (p.p.)*
1. Examen físico documentado en el momento de la admisión.	25/278 (9,0%)	63/134 (47,1%)	+38,1
2. Documentación del historial de hábitos de salud (por ejemplo, tabaquismo, consumo de alcohol, consumo de drogas, dieta, sueño y actividad física).	12/278 (4,3%)	6/134 (4,5%)	+0,2
3. Seguimiento de los efectos secundarios de los antipsicóticos.	52/270 (19,2%)	8/131 (6,1%)	–13,1
4. Programación de citas de seguimiento.	3/278 (1,1%)	29/134 (21,6%)	+20,5
5. Análisis de sangre en ayunas en el último año.	4/278 (1,4%)	0/134 (0,0%)	–1,4
6. Electrocardiograma documentado en el último año.	2/278 (0,7%)	0/134 (0,0%)	–0,7
7. Peso, altura y/o índice de masa corporal registrados en los últimos seis meses.	0/278 (0,0%)	30/134 (22,4%)	+22,4

*: Δ (p.p.) = Diferencia en puntos porcentuales entre el inicio y el seguimiento. **: Para el criterio 3, hubo una ligera diferencia en la población elegible (línea de base N = 270; seguimiento N = 131) debido a la prescripción de medicamentos antipsicóticos.

## DISCUSIÓN

El objetivo de este proyecto de implementación era armonizar las prácticas de evaluación y detección de la salud física en un centro comunitario de salud mental brasileño con las recomendaciones sobre buenas prácticas. El objetivo era mejorar la identificación, documentación y gestión de las afecciones de salud física entre las personas con SPMD, que se sabe que padecen tasas desproporcionadamente altas de enfermedades prevenibles y mortalidad prematura^(18,19)^. Al integrar procesos de atención médica física basados en la evidencia en los servicios de salud mental de rutina, el proyecto pretendía abordar una deficiencia significativa que ha sido ampliamente documentada en la literatura nacional e internacional.

La auditoría inicial confirmó esta deficiencia. El cumplimiento de los siete criterios basados en la evidencia fue alarmantemente bajo, oscilando entre el 0% y el 20,3%. Estos hallazgos concuerdan con investigaciones anteriores que muestran que las personas con SPMD a menudo reciben una atención médica física inadecuada debido a la fragmentación sistémica, la baja prioridad que se le da a la salud física en los entornos psiquiátricos y las barreras a nivel de los proveedores, como el estigma, la ambigüedad de roles y la falta de capacitación^(5,20,21)^. En este caso, la auditoría sirvió para cuantificar el alcance del problema, crear conciencia entre el personal y proporcionar un punto de partida concreto para la intervención.

Tras la implementación de estrategias específicas, la auditoría de seguimiento reveló mejoras significativas en tres de los indicadores prioritarios. El porcentaje de pacientes que recibieron una evaluación de salud física en el momento de su ingreso aumentó del 9% al 47,1% (criterio 1); el porcentaje de pacientes con citas de seguimiento programadas aumentó del 1.1% al 21.6% (criterio 4); y el porcentaje de pacientes con peso, estatura o índice de masa corporal documentados mejoró del 0% al 22.4% (criterio 7). Estos resultados ponen de relieve el potencial de las estrategias sencillas y pragmáticas para mejorar la calidad de la atención en entornos con recursos limitados.

En particular, la introducción de un sello estandarizado durante las evaluaciones iniciales permitió a las enfermeras documentar sistemáticamente los parámetros de salud física como parte de su flujo de trabajo establecido. La reconfiguración de las funciones profesionales para involucrar más activamente a las enfermeras en las evaluaciones físicas también resultó eficaz para promover la atención en equipo. Las mejoras en la programación de los seguimientos sugieren la aparición de una cultura de continuidad de la atención, un paso fundamental en el monitoreo y el manejo a largo plazo de las enfermedades crónicas que a menudo se pasa por alto en los servicios psiquiátricos. Del mismo modo, el aumento de la documentación de los datos antropométricos es un enfoque fundamental y vital para identificar a las personas con riesgo de complicaciones metabólicas, especialmente cuando el acceso a las pruebas de laboratorio es limitado^(22)^.

Estas mejoras son coherentes con la amplia bibliografía sobre la ciencia de la implementación, que ha demostrado que las intervenciones diseñadas y puestas en práctica conjuntamente por los trabajadores de primera línea tienden a ser más aceptables, viables y sostenibles^(23,24)^. Cuando el personal participa en la definición de los problemas y las soluciones, es más probable que se apropie de los cambios, los adapte a las circunstancias locales y se asegure de que se cumplan en la práctica diaria^(23)^.

No obstante, los avances variaron en función de los criterios. Dos indicadores mostraron poca o ninguna mejora. El criterio 2 (documentación exhaustiva del historial de comportamientos relacionados con la salud) aumentó del 4,3% al 4,5%. Este indicador requería el registro de múltiples comportamientos relacionados con la salud, como el consumo de tabaco, alcohol y drogas ilegales, la dieta, el sueño y la actividad física. Sin embargo, en muchos casos solo se documentó un subconjunto de estos comportamientos. Esto sugiere que el alcance del indicador puede haber excedido lo que era factible con las rutinas de documentación existentes. Esto pone de relieve la necesidad de herramientas o listas de verificación estandarizadas para facilitar la recolección de datos exhaustivos sin sobrecargar al personal.

El porcentaje de pacientes cuyos efectos secundarios antipsicóticos se monitorearon disminuyó, pasando del 19.2% al 6.1%. Esta disminución puede deberse a la limitada participación de los médicos prescriptores en el proceso de implementación y a la ausencia de formatos estandarizados o indicaciones para registrar la información sobre los efectos secundarios. Es posible que los médicos optaran por no documentar nada cuando no se notificaban efectos secundarios; sin embargo, las mejores prácticas hacen hincapié en que se debe monitorear de forma sistemática, independientemente de si se observan efectos adversos^(25)^. Para abordar esta cuestión se necesitarán estrategias más adaptadas que refuercen la participación y la responsabilidad de los médicos en las iniciativas de implementación. Esto podría implicar el desarrollo conjunto de formularios de monitoreo estandarizados, la incorporación de indicaciones en los registros clínicos, la realización de sesiones de retroalimentación específicas para los prescriptores y la vinculación del monitoreo de los antipsicóticos con planes de atención multidisciplinarios.

No se observó ninguna mejora en los criterios 5 (pruebas de laboratorio, que descendieron del 1,4% al 0%) y 6 (realización de electrocardiogramas, que descendieron del 0,7% al 0%), ambos muy dependientes de servicios externos. Estos procedimientos implican múltiples pasos logísticos, como la emisión de solicitudes de pruebas, la programación y la asistencia a citas en laboratorios municipales, la obtención de resultados y el regreso al CAPS para el seguimiento. Dado el corto plazo entre las auditorías, no es de extrañar que no se observaran cambios significativos en estos indicadores. Sin embargo, esto pone de relieve un problema estructural más amplio ya documentado en la literatura: la limitada integración entre los servicios comunitarios de salud mental y el sistema de salud en general limita gravemente la viabilidad de una atención oportuna y coordinada^(23)^. Sin sistemas interoperables, vías de derivación más claras y un mejor acceso a los servicios, incluso las iniciativas internas bien planificadas pueden no alcanzar su impacto potencial^(26,27)^.

La falta de mejora en los indicadores que dependen de servicios externos pone de relieve que las barreras a la atención de salud física pueden extenderse más allá del ámbito de los servicios especializados de salud mental. En este contexto, otra consideración importante es cómo se conectan las diferentes partes de la red de atención de salud. Las dificultades para integrar los servicios pueden conducir a la fragmentación de la red de atención, lo que limita la prestación de una atención longitudinal y integral^(28)^. Un estudio mostró que los gestores, los profesionales de la atención de salud y los pacientes identificaban como principales barreras la falta de vías de atención claramente definidas, el conocimiento limitado de los componentes de la red y los criterios de acceso, y las dificultades en la comunicación de los casos y el alta de los servicios especializados^(28)^. En este contexto, los modelos de atención compartida, como el apoyo matricial, fomentan la colaboración entre los servicios y promueven un enfoque integrado y coordinado para abordar las necesidades de salud mental y física^(29)^.

Deben tenerse en cuenta algunas limitaciones. En primer lugar, el plazo relativamente corto entre la evaluación inicial en diciembre de 2024 y la evaluación de seguimiento en marzo de 2025 puede no haber permitido la implementación y consolidación completas de los cambios. Las mejoras observadas pueden reflejar en parte el efecto Hawthorne, más que un cambio de comportamiento sostenido. En segundo lugar, no se registraron sistemáticamente los datos sobre las medidas del proceso y la fidelidad, como la proporción de evaluaciones en las que se utilizó el sello estandarizado, el cumplimiento de las prácticas de documentación entre los profesionales y el número de reuniones de retroalimentación realizadas. La ausencia de estos indicadores limita la capacidad de interpretar con mayor precisión el proceso de implementación y sus mecanismos de cambio. En tercer lugar, el menor tamaño de la muestra en la auditoría de seguimiento, debido a la rotación de usuarios y a las variaciones en el flujo de servicios, puede afectar a la comparabilidad.

Por último, dado que la evaluación se basó únicamente en la revisión de los expedientes médicos, es posible que se hayan subestimado los cambios reales en la práctica clínica cuando la documentación estaba incompleta o se retrasaba. Sin embargo, estas limitaciones son comunes en las iniciativas de mejora de la calidad en el mundo real y ponen de relieve la importancia de incluir la evaluación de procesos y la triangulación de fuentes de datos en futuros estudios.

A pesar de estas limitaciones, el proyecto generó conocimientos prácticos para mejorar el monitoreo de la salud física en los servicios comunitarios de salud mental en Brasil y otros contextos similares. Nuestros resultados demuestran que las intervenciones impulsadas por equipos, adaptadas a los flujos de trabajo locales y respaldadas por ayudas visuales o herramientas sencillas, son viables y eficaces. Los esfuerzos futuros deben dar prioridad a la integración entre servicios, mejorar la coherencia de la documentación clínica e incorporar ciclos estructurados de auditoría y retroalimentación en la práctica habitual.

Además, la inclusión de las perspectivas de los usuarios en los estudios que evalúan los resultados a largo plazo podría profundizar nuestra comprensión de cómo la mejora de las pruebas de detección se traduce en una mejor salud física y mental. En última instancia, satisfacer las necesidades de salud física de las personas con enfermedades mentales graves requiere un compromiso institucional sostenido, una responsabilidad compartida y una visión más amplia de la atención integrada y equitativa, así como soluciones técnicas^(27)^.

## CONCLUSIÓN

Este proyecto demostró que la incorporación de estrategias sencillas y de bajo costo en los flujos de trabajo rutinarios puede mejorar significativamente las prácticas de detección de la salud física en los entornos comunitarios de salud mental. La introducción de herramientas de documentación estandarizadas y la redefinición de las funciones profesionales —en particular, la mayor participación del personal de enfermería— dieron lugar a mejoras significativas en áreas clave como las evaluaciones físicas, las mediciones antropométricas y la programación de citas de seguimiento. Estos cambios señalan un avance inicial hacia la integración de la atención física y mental.

Sin embargo, el proyecto también puso de relieve las limitaciones de la participación parcial del equipo y la fragmentación estructural, ya que los indicadores que dependen de la participación de los médicos o de la coordinación de servicios externos mostraron poca o ninguna mejora. Las iniciativas futuras deben basarse en estas lecciones, fomentando una planificación inclusiva, reforzando la colaboración interprofesional y abordando las barreras sistémicas que impiden la continuidad de la atención. Los próximos pasos prácticos incluyen el fortalecimiento del vínculo entre CAPS II, la atención primaria y los servicios de laboratorio para facilitar el seguimiento de los parámetros clínicos, la implementación de listas de verificación estandarizadas para monitorear los efectos secundarios de los antipsicóticos y la integración de recordatorios o avisos electrónicos en los registros de salud para apoyar la documentación rutinaria. Las mejoras sostenidas también dependerán de la institucionalización de mecanismos de auditoría y retroalimentación, así como del desarrollo profesional continuo. Al ilustrar el potencial y los retos de la mejora de la atención de salud física en los entornos de atención psicosocial, este estudio proporciona información útil para los sistemas de salud que tienen como objetivo reducir la brecha de mortalidad que sufren las personas con trastornos mentales graves.

## DISPONIBILIDAD DE DATOS

El conjunto de datos que respaldan las conclusiones de este estudio está disponible previa solicitud al autor correspondiente.
